# Fast food consumption and risk of non-alcoholic fatty liver disease: a systematic review and meta-analysis

**DOI:** 10.3389/fpubh.2025.1600826

**Published:** 2025-07-30

**Authors:** Jinke He, Yingxue Wang, Fangbin Weng

**Affiliations:** ^1^Department of Infectious Disease, Yiwu Central Hospital, Zhejiang, China; ^2^Department of Public Health and Geriatric Health Guidance, Yiwu Central Hospital, Zhejiang, China

**Keywords:** non-alcoholic fatty liver disease, fast food, obesity, meta-analysis, systematic review

## Abstract

**Background:**

Non-alcoholic fatty liver disease (NAFLD) is a global health issue, with fast food consumption hypothesized as a risk factor. This meta-analysis aimed to explore the relationship between fast food intake and NAFLD.

**Methods:**

This review was conducted according to the Preferred Reporting Items for Systematic Reviews and Meta-Analyses (PRISMA) 2020 guidelines. A comprehensive search was conducted across PubMed, Web of Science, Scopus, and Embase from inception to February 28, 2025. A total of nine eligible observational studies involving 169,771 participants were included. Pooled odds ratios (ORs) with 95% confidence intervals (CIs) were calculated using random-effects models.

**Results:**

A higher consumption of fast food was significantly associated with a 55% increased risk of NAFLD (OR = 1.55, 95% CI: 1.51–1.59, *p* < 0.001, I^2^ = 15.6%). Moreover, fast food intake was linked to a 37% higher risk of obesity (OR = 1.37, 95% CI: 1.27–1.49, *p* < 0.001, I^2^ = 54.2%), a key metabolic factor in NAFLD pathogenesis. Sensitivity analysis confirmed the robustness of these associations, with no significant evidence of publication bias.

**Conclusion:**

Fast food consumption is positively associated with NAFLD and obesity. Heterogeneity highlights the need for standardized methods in future large-scale studies to validate these findings and inform preventive strategies.

## 1 Introduction

Non-alcoholic fatty liver disease (NAFLD), the most prevalent chronic liver condition worldwide, presents a rising burden on global health systems (1). With a global prevalence of up to 25% and a marked increase in metabolic syndrome populations, NAFLD poses a significant burden on healthcare systems (1). Characterized by excessive hepatic triglyceride accumulation, this disease can progress insidiously to non-alcoholic steatohepatitis (NASH), liver fibrosis, and hepatocellular carcinoma, underscoring its clinical relevance (2). Dietary patterns have shifted significantly in recent decades, with fast food consumption rising exponentially due to its accessibility, affordability, and convenience (2, 3). This dietary shift is particularly concerning, as fast food consumption patterns are typically rich in energy-dense, hyperpalatable components (e.g., saturated fats, refined sugars, and sodium) while lacking essential nutrients, potentially exacerbating metabolic dysfunction (3).

Although robust biological mechanisms link fast food intake to NAFLD pathogenesis—including induction of insulin resistance, promotion of visceral adiposity, and perturbation of gut microbiota—epidemiological evidence remains inconsistent (4, 5). Observational studies have reported conflicting results, with some demonstrating a positive dose-dependent association between fast food consumption and NAFLD risk (4), while others found no significant correlation (5). These discrepancies may stem from methodological heterogeneity, such as differences in dietary assessment tools (e.g., self-reported questionnaires vs. objective biomarkers) and population demographics (age, ethnicity, and baseline metabolic status).

To address the inconsistencies in the literature, we performed a systematic review and meta-analysis to examine the association between fast food consumption and NAFLD risk. By synthesizing data from high-quality observational studies, we aimed to assess the overall impact of fast food intake on NAFLD prevalence.

## 2 Materials and methods

### 2.1 Search strategy

A systematic literature search was conducted across four electronic databases (PubMed, Web of Science, Scopus, and Embase) from inception to February 28, 2025, to identify all relevant studies evaluating the association between fast food consumption and NAFLD. The search strategy combined medical subject headings (MeSH terms) and free-text keywords related to both exposures and outcomes. For NAFLD, the search included terms such as “non-alcoholic fatty liver disease,” “NAFLD,” “steatohepatitis,” and “hepatic steatosis.” For fast food consumption, terms included “fast food,” “junk food,” “takeaway meals,” and “processed food intake.” Boolean operators (AND/OR) were used to combine these concepts, with truncation and wildcards applied to capture variant spellings (e.g., “consum*” for consumption/consumer). Database-specific adjustments were made to accommodate syntax differences. For example, in PubMed, MeSH terms were exploded (e.g., “fast food” [Mesh] OR “fast food” [tiab]) and combined with free-text terms. In Scopus, adjacency operators (e.g., “fast food” NEAR/3 “consum*”) were used to enhance precision. A full search syntax for each database is provided in Table 1.

**Table 1:** Search strategy in this meta-analysis.

Database	Search strategy
Pubmed	(“fast food” [Title/Abstract] OR “junk food” [Title/Abstract] OR “Western diet” [Title/Abstract] OR “processed food” [Title/Abstract]) AND (“non-alcoholic fatty liver disease” [Title/Abstract] OR “NAFLD” [Title/Abstract] OR “hepatic steatosis” [Title/Abstract] OR “fatty liver” [Title/Abstract])
Embase	(“fast food”: ab,ti OR “junk food”: ab,ti OR “Western diet”: ab,ti OR “processed food”: ab,ti) AND (“non-alcoholic fatty liver disease”: ab,ti OR “NAFLD”: ab,ti OR “hepatic steatosis”: ab,ti OR “fatty liver”: ab,ti)
Scopus	TITLE-ABS-KEY (“fast food” OR “junk food” OR “Western diet” OR “processed food”) AND TITLE-ABS-KEY (“non-alcoholic fatty liver disease” OR “NAFLD” OR “hepatic steatosis” OR “fatty liver”)
Web of science	TS = (“fast food” OR “junk food” OR “Western diet” OR “processed food”) AND TS = (“non-alcoholic fatty liver disease” OR “NAFLD” OR “hepatic steatosis” OR “fatty liver”)

### 2.2 Eligibility criteria

#### 2.2.1 Study design

Only observational studies were eligible for inclusion in this systematic review and meta-analysis. This specifically encompassed cohort studies, which follow a group of individuals over time to observe the development of outcomes related to exposure; case–control studies, which compare individuals with a particular outcome (cases) to those without it (controls) to investigate the association with prior exposures; and cross-sectional studies, which assess the exposure and outcome simultaneously in a defined population at a single point in time.

#### 2.2.2 Language

All publications considered for inclusion had to be in the English language. This language restriction was applied to ensure accurate interpretation and consistent quality assessment of the included studies. While this may introduce a potential language bias, it was deemed necessary due to resource limitations and to minimize the risk of data misinterpretation.

#### 2.2.3 Population

The studies had to involve individuals with or without a diagnosis of NAFLD. This broad population scope was chosen to comprehensively assess the relationship between fast food consumption and NAFLD. By including both groups, it was possible to examine the impact of fast food intake on the development of NAFLD in those without the disease and any potential associations with disease severity or progression in those already diagnosed. Studies that focused solely on populations with other liver diseases or conditions unrelated to NAFLD, or those that did not provide any information on the presence or absence of NAFLD, were excluded.

#### 2.2.4 Data availability

In this meta-analysis, fast food consumption is defined based on either of the following criteria: (1) a frequency-based definition, where individuals consume fast food more than three times per week, or (2) a proportion-based definition, where fast food accounts for at least 20% of the total dietary intake. NAFLD is characterized by lipid accumulation, primarily triacylglycerol, in hepatocytes of individuals with minimal alcohol intake (≤2 drinks/day for women, ≤3 drinks/day for men), after excluding other causes of steatosis, such as chronic liver diseases (hepatitis A, B, and C, Wilson’s disease) or medication-induced effects. Studies needed to provide data on the frequency or amount of fast food intake and a corresponding diagnosis of NAFLD. For fast food consumption, this could include details such as the number of times fast food was consumed per week or month, the portion sizes, or any other measure that quantified the exposure. The diagnosis of NAFLD is based on the “Guidelines for the Prevention and Treatment of Metabolic Dysfunction-Associated (Non-Alcoholic) Fatty Liver Disease (Version 2024)” issued by the Chinese Society of Hepatology (6). Studies that lacked sufficient data on either fast food consumption or NAFLD diagnosis were not eligible for inclusion.

### 2.3 Study selection

Two independent reviewers meticulously screened the search results. First, they reviewed the titles and abstracts of all retrieved studies to identify potentially relevant ones. Studies that clearly did not meet the eligibility criteria were excluded at this stage. Subsequently, the full-text versions of the remaining potentially relevant studies were obtained and carefully evaluated. Any discrepancies in the selection process between the two reviewers were resolved through in-depth discussion. If necessary, a third reviewer was consulted to reach a consensus.

### 2.4 Data extraction

Once the eligible studies were selected, the two independent reviewers extracted the relevant data from each study. Any discrepancies in data extraction were resolved through discussion. If a consensus could not be reached, a third reviewer was consulted to adjudicate and finalize the extracted information. The data extracted included basic study characteristics such as the study design, year of publication, country of origin, sample size, age range of the participants, and ethnic composition. Regarding the exposure variable (fast food consumption), details about how it was measured (e.g., self-reported questionnaires with specific recall periods like weekly or monthly intake, objective measures such as food frequency records), and the actual values or categories of fast food intake were noted.

### 2.5 Quality assessment

The quality of each included study was evaluated using the Newcastle-Ottawa Scale (NOS) (7). The NOS assesses three main aspects of observational studies: selection of the study population, comparability of the groups, and ascertainment of the outcome. For cohort studies, points are assigned based on factors such as the representativeness of the exposed cohort, the selection of the non-exposed cohort, and the adequacy of follow-up. In case–control studies, aspects like the definition of cases and controls, the selection of controls, and the ascertainment of exposure are considered. For cross-sectional studies, the sampling method, the definition of the population, and the assessment of exposure and outcome are evaluated. Each study was given a score on a scale of 0–9, with higher scores indicating better-quality studies. To enhance transparency and allow detailed evaluation of study quality, the full NOS scoring for each included study is provided in Supplementary File 1.

### 2.6 Statistical analysis

For dichotomous outcomes (presence or absence of NAFLD), odds ratios (ORs) with 95% confidence intervals (CIs) were calculated. These ORs were used to estimate the strength of the association between fast food consumption and the risk of NAFLD. Heterogeneity was quantified using the Cochrance Q statistic and the I^2^ statistic. The Cochrance Q statistic tests the null hypothesis that all studies are estimating the same effect size, while the I^2^ statistic measures the proportion of total variation in study estimates that is due to heterogeneity rather than chance. A value of I^2^ > 50% and a significant Cochrance Q statistic (*p* < 0.05) were considered indicative of substantial heterogeneity. Sensitivity analyses were also conducted by sequentially removing each study from the meta-analysis to assess the stability of the overall results.

## 3 Results

### 3.1 Basic characteristics of the included studies

**Figure 1:**
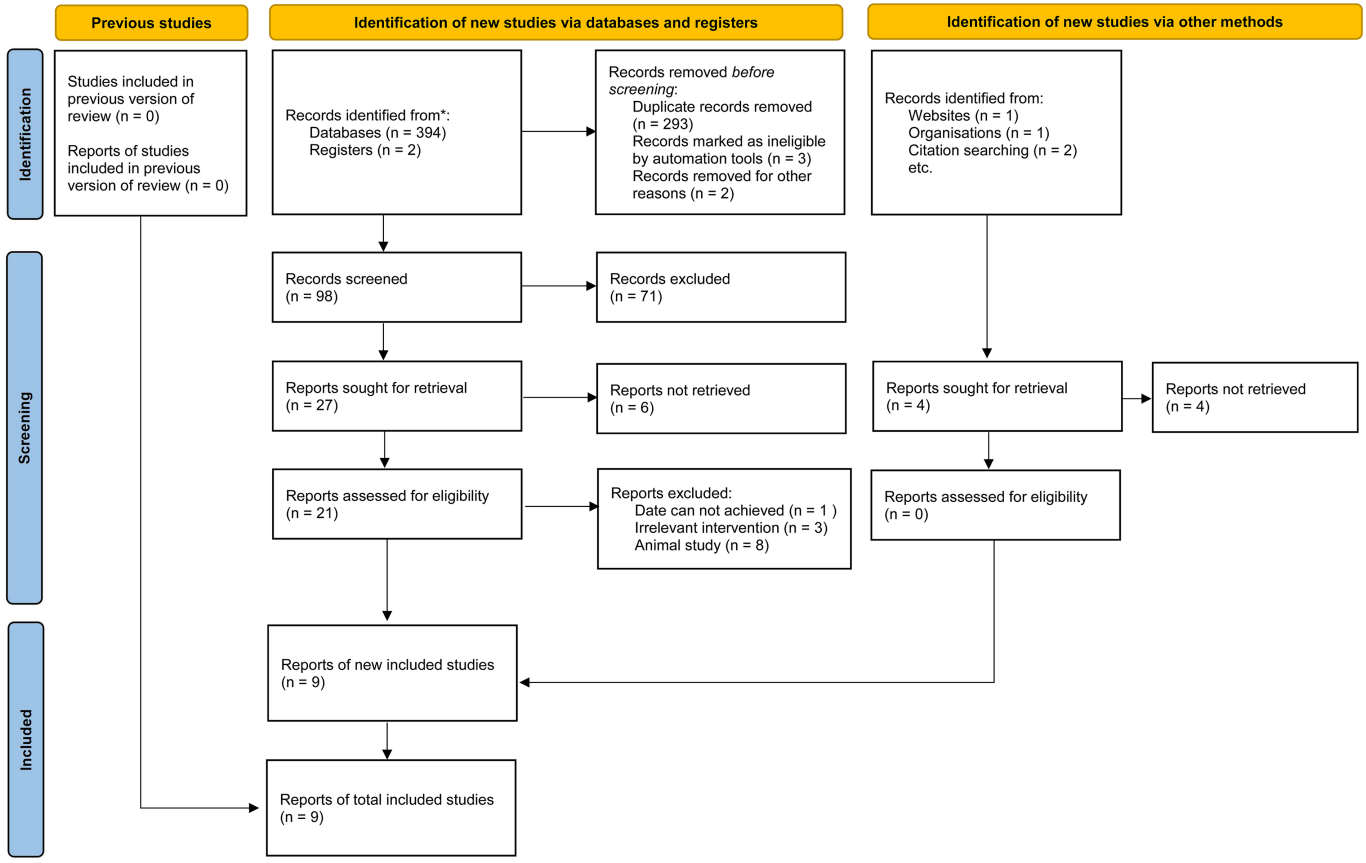
PRISMA 2020 flow diagram.

This meta-analysis included nine studies conducted across diverse geographical regions, including Lebanon, Spain, Greece, the USA, Bangladesh, Iran, and the UK (8–16). The study designs varied, comprising case–control (*n* = 3), cross-sectional (*n* = 3), longitudinal (*n* = 1), and prospective cohort (*n* = 2) methodologies. The sample sizes ranged from 46 to 86,944 participants, with a total of 169,771 individuals analyzed. Participants were categorized into fast food and non-fast food diet groups. The mean age ranged from 17.2 to 56.0 years, and the proportion of male participants varied across studies, with some reporting a near-equal gender distribution while others exhibited a male predominance. Several studies also assessed obesity and body mass index (BMI) differences between dietary groups, consistently reporting higher BMI or a greater prevalence of obesity among fast food consumers. The methodological quality of the included studies, as assessed by the NOS, ranged from 7 to 9, indicating a generally high level of study rigor. The literature screening process adhered to PRISMA_2020 guidelines (Figure 1), and the basic information of the included studies is presented in Table 2. To ensure transparency and compliance with systematic review reporting standards, the completed PRISMA 2020 checklist has been provided as Supplementary File 2.

**Table 2:** Basic characteristics of included studies.

First author, year	Country	Study design	Sample size (*n*)	Diet group (*n*, fast food vs. non-fast food)	Age (years, fast food vs. non-fast food)	Male (*n*, fast food vs. non-fast food)	NAFLD (*n*, fast food vs. non-fast food) and NAFLD diagnosis method (e.g., ultrasound, MRI, CAP, ICD codes)	Obesity (*n*, fast food vs. non-fast food)	Key findings	NOS score
Fakhoury-Sayegh et al., 2017 (8)	Lebanon	Case–control	222	18 vs. 15	39.9 ± 6.0 vs. 38.8 ± 13.2	55 vs. 44	112 vs. 110, Ultrasound	Obesity: 62 vs. 9	The study identified a fast food dietary pattern (rich in red meat, hamburgers, fries, carbonated drinks) as a key risk factor for NAFLD, increasing odds by 4-fold.	8
García et al., 2025 (9)	Spain	Longitudinal	46	23 vs. 23	52.4 ± 6.5 vs. 50.8 ± 6.9	13 vs. 15	16 vs. 9, MRI and ultrasonography	–	Reducing fast food intake significantly lowered intrahepatic fat content (IFC) by 7.7% in the group with the largest UPF reduction.	9
Kalafati et al., 2019 (10)	Greece	Case–control	351	86 vs. 73	50.4 ± 10.5 vs. 43.8 ± 11.2	61 vs. 85	134 vs. 217, Ultrasonography	Obesity: 103 vs. 146	A fast food-type diet (high in energy-dense, sugary, and saturated fat foods) was associated with a 3.9-fold increased NAFLD risk, linked to higher CRP and uric acid. Conversely, the unsaturated fatty acid diet (nuts, chocolate) reduced NAFLD odds by 55.7% in the second quartile.	7
Kardashian et al., 2023 (11)	USA	Cross-sectional	3,954	1,147 vs. 2,807	42.0 ± 5.2 vs. 50.0 ± 4.8	607 vs. 1,319	267 vs. 263, Vibration-controlled transient elastography (CAP ≥263 dB/m)	Obesity: 574 vs. 1,123	Fast food consumptio*n* (≥20% of daily calories) was associated with 1.45-fold increased NAFLD odds in U. S. adults.	8
Liu et al., 2023 (12)	USA	Cross-sectional	6,545	1,538 vs. 1982	53.6 ± 5.5 vs. 47.2 ± 3.4	1,165 vs. 1862	2,224 vs. 4,321, US fatty liver index ≥30	Obesity: 1325 vs. 1890	Higher fast food intake was associated with a 1.83-fold increased NAFLD risk in US adults.	7
Saha et al., 2022 (16)	Bangladesh	Cross-sectional	174	111 vs. 63	17.3 ± 0.8 vs. 17.2 ± 0.7	62 vs. 37	76 vs. 25, Ultrasonography	–	Fast food consumers facing double the NAFLD risk (adjusted odds ratio) compared to non-consumers.	8
Talenezhad et al., 2022 (13)	Iran	Case–control	240	120 vs. 120	44.2 ± 10.4 vs. 43.5 ± 12.1	50 vs. 45	115 vs. 102, Ultrasonography	Obesity: 92 vs. 69	A western dietary pattern (high in fast food) was associated with a 3.52-fold increased NAFLD risk in Iranian adults.	7
Zhang et al., 2024 (14)	UK	Prospective cohort	71,295	35,337 vs. 35,958	53.1 ± 8.1 vs. 56.0 ± 7.6	15,696 vs. 15,807	583 vs. 389, ICD-10 (K75.8, K76.0)	Obesity: 17668 vs. 17,582	Higher fast food intake was associated with a 9% increased risk of severe NAFLD per 10% intake increment, with the highest quartile showing a 26% elevated risk.	8
Zhao et al., 2024 (15)	UK	Prospective cohort	86,944	43,472 vs. 43,472	54.9 ± 8.2 vs. 56.2 ± 7.8	1,9,859 vs. 22,156	415 vs. 208, ICD-10 (K75.8, K76.0)	Obesity: 12,651 vs. 7,122	Higher fast food intake was associated with 1.43-fold increased NAFLD risk.	7

BMI, body mass index; CAP, Controlled Attenuation Parameter; ICD-10, International Classification of Diseases, 10th Revision; MRI, Magnetic Resonance Imaging; NAFLD, Non-Alcoholic Fatty Liver Disease; NOS, Newcastle–Ottawa Scale. “–” indicates information not reported or not applicable in the original publication. The NOS score ranges from 0 to 9, with higher scores indicating better methodological quality.

### 3.2 Association between fast food consumption and the risk of NAFLD

**Figure 2:**
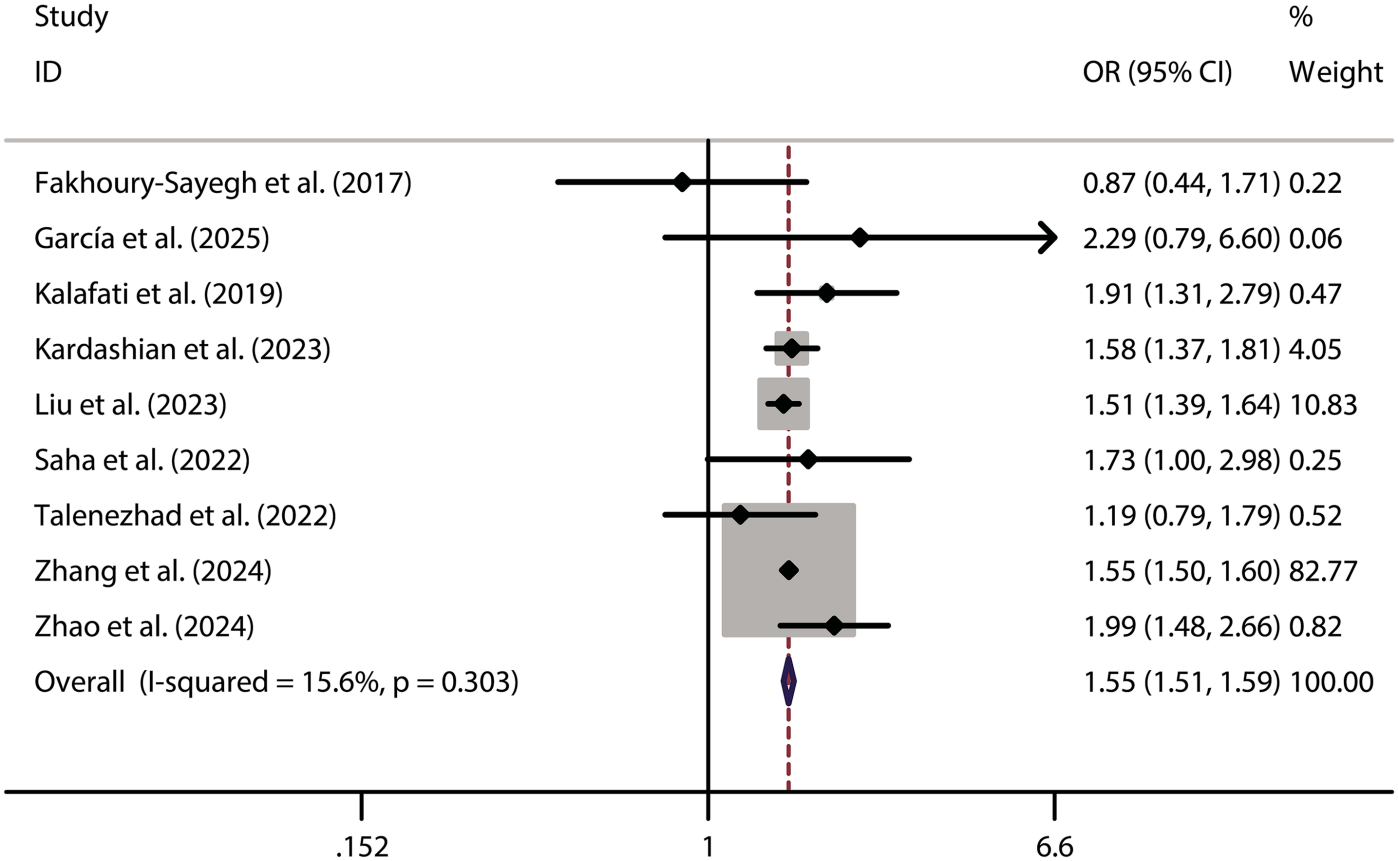
Forest plot of fast food consumption and NAFLD risk.

**Figure 3:**
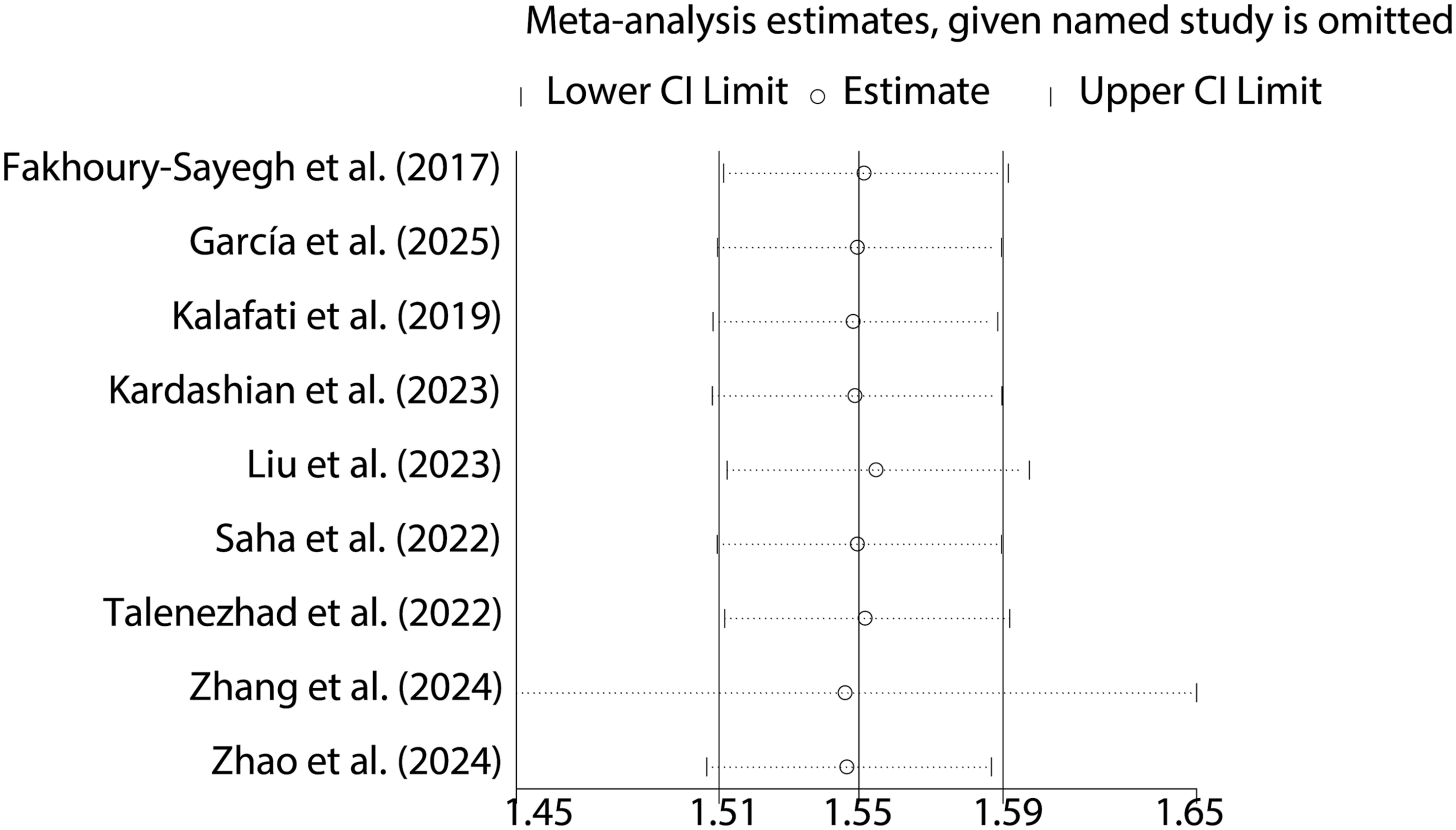
Sensitivity analysis for NAFLD risk.

**Figure 4:**
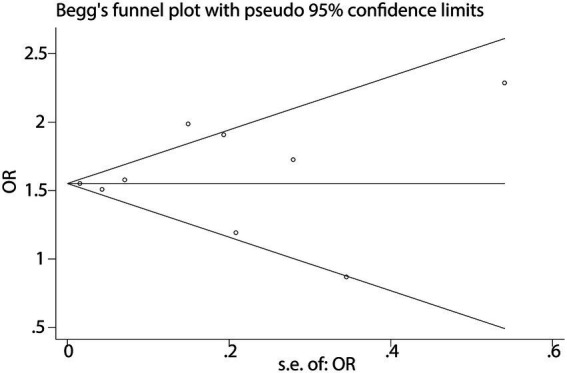
Funnel plot for publication bias in NAFLD studies.

When analyzing the dichotomous outcome of NAFLD presence or absence, the pooled results from our meta-analysis demonstrated that increased fast food consumption is significantly associated with a higher risk of developing NAFLD. The combined OR was 1.55 (95% CI: 1.51–1.59, *p* < 0.001, I^2^ = 15.6%, Figure 2). Sensitivity analysis confirmed the robustness of these findings (Figure 3), and publication bias assessment suggested no significant bias affecting the results (Figure 4). These findings highlight the potential role of dietary patterns in NAFLD risk and emphasize the need for dietary interventions to mitigate this risk.

### 3.3 Association between fast food consumption and the risk of obesity

**Figure 5:**
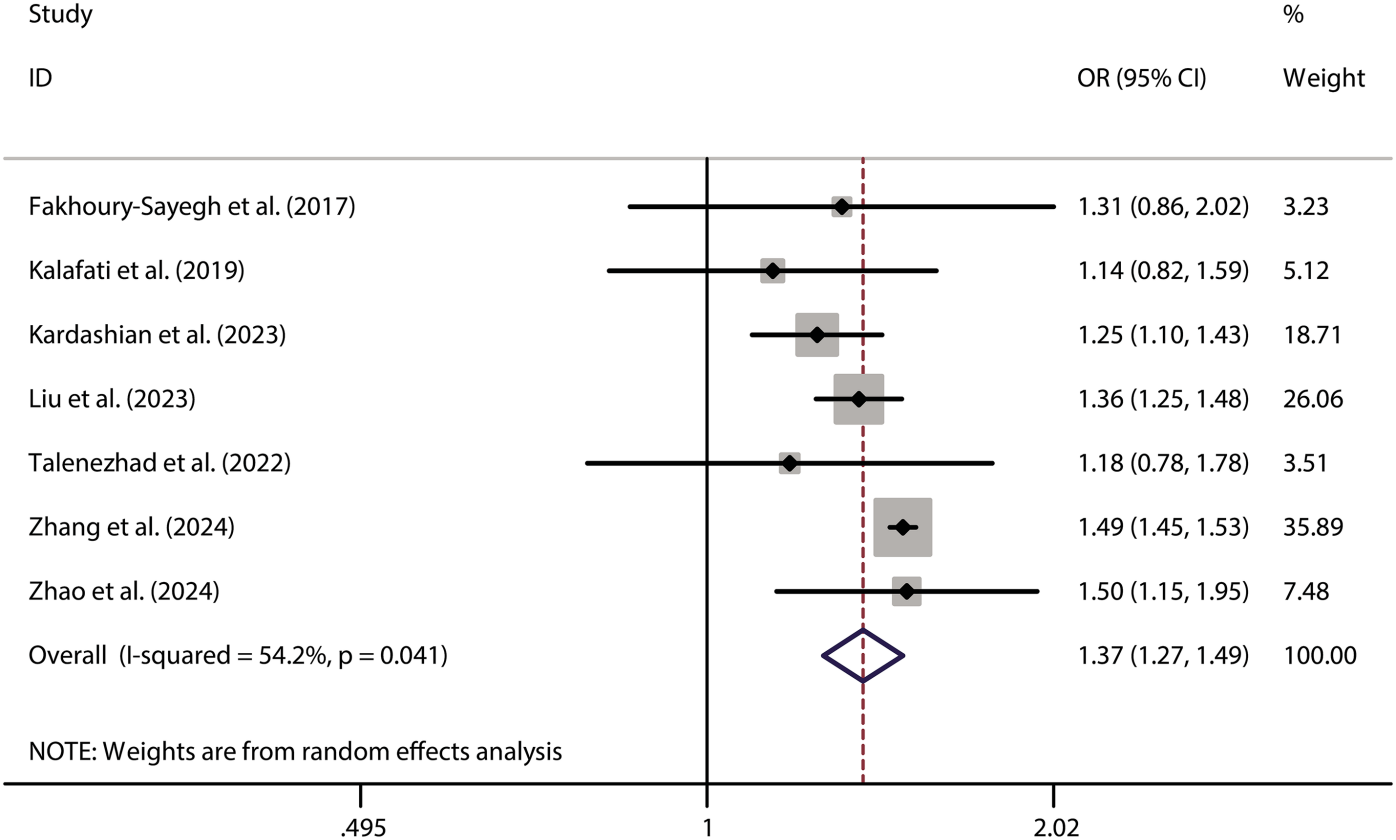
Forest plot of fast food consumption and obesity risk.

**Figure 6:**
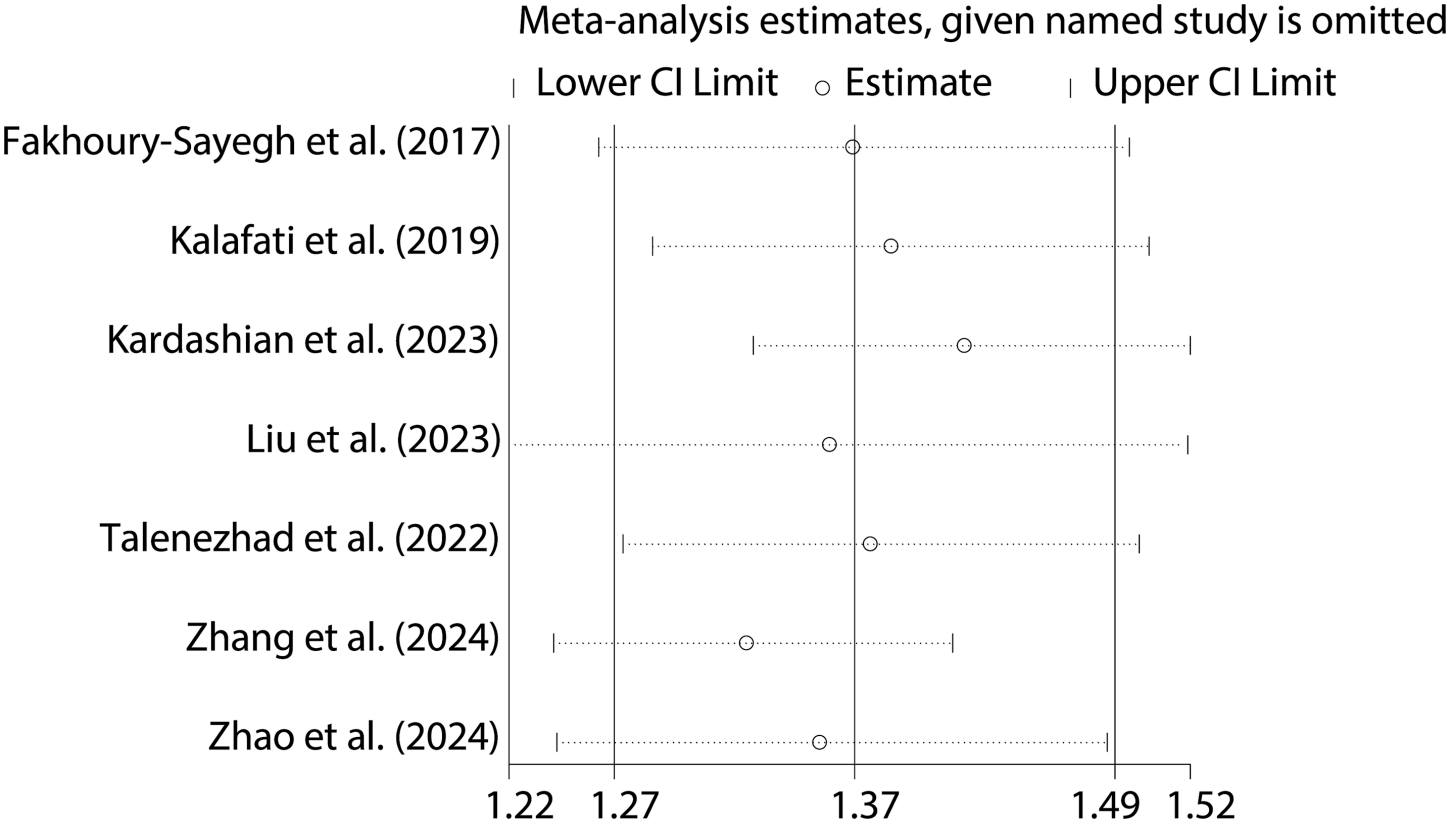
Sensitivity analysis for obesity risk.

**Figure 7:**
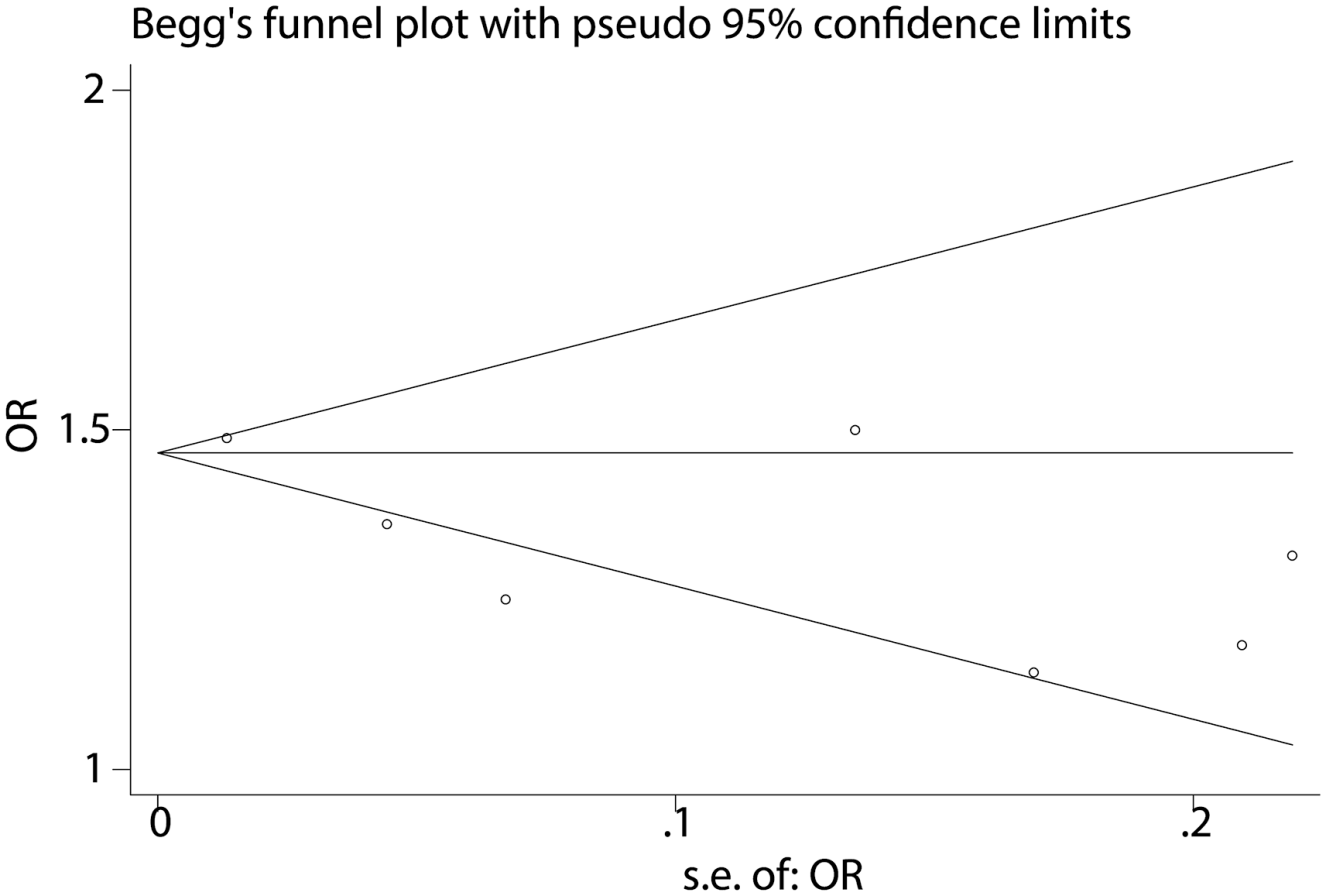
Funnel plot for publication bias in obesity studies.

To further validate the relationship between fast food consumption and fat accumulation, we conducted a meta-analysis of seven studies that examined the association between fast food intake and obesity. The pooled results indicated that fast food consumption is a significant risk factor for obesity, with a combined OR of 1.37 (95% CI: 1.27–1.49, *p* < 0.001, I^2^ = 54.2%, Figure 5). Sensitivity analysis (Figure 6) confirmed the robustness of these findings, and no significant publication bias was detected (Figure 7).

## 4 Discussion

NAFLD has emerged as a global health crisis, affecting approximately 25% of the population and posing significant risks for cirrhosis and hepatocellular carcinoma (17). This epidemic has coincided with a dramatic surge in fast food consumption, which is characterized by its high content of saturated fats, refined sugars, and sodium, coupled with easy accessibility and affordability (18). Mechanistically, fast food diets promote insulin resistance, visceral adiposity, and gut microbiota dysbiosis, all of which are implicated in NAFLD pathogenesis (19). However, epidemiological studies have yielded conflicting results, with some reporting a positive association between fast food intake and NAFLD (20), while others found no significant link (21). These inconsistencies likely stem from methodological variations in dietary assessment, NAFLD diagnostic criteria, and population demographics. To address this ambiguity, we conducted a systematic review and meta-analysis to quantify the association between fast food consumption and NAFLD, identify sources of heterogeneity, and inform future research.

Our meta-analysis of 9 observational studies revealed a 55% increased risk of NAFLD among individuals with higher fast food consumption (OR = 1.55, 95% CI: 1.51–1.59, *p* < 0.001). This finding aligns with biological plausibility, as fast food diets are known to induce hepatic steatosis through multiple pathways: (1) insulin resistance: high intake of refined sugars and trans fats impairs insulin signaling, leading to increased lipolysis and hepatic fat accumulation (22). (2) Obesity: fast food consumption was associated with a 37% higher obesity risk (OR = 1.37, 95% CI: 1.27–1.49), and obesity is a key driver of NAFLD via adipose tissue inflammation and ectopic fat deposition (3). (3) Gut microbiota dysbiosis: processed foods may alter gut microbial composition, promoting endotoxemia and hepatic inflammation (5).

Despite robust findings, several limitations warrant consideration: (1) Heterogeneity: the I^2^ statistic for NAFLD (15.6%) indicates low heterogeneity, suggesting consistent findings across studies evaluating this outcome. In contrast, the I^2^ for obesity (54.2%) suggests moderate heterogeneity. This may arise from multiple sources. First, differences in NAFLD diagnostic criteria (e.g., ultrasound vs. MRI vs. CAP or ICD codes) may lead to inconsistent outcome classification. Second, regional dietary habits could influence both the type and frequency of fast food consumed—fast food in Mediterranean countries (e.g., Spain, Greece) often differs in composition and preparation methods compared to Western or Asian settings, potentially moderating its metabolic impact. Third, population characteristics such as age ranges (e.g., adolescents vs. older adults), genetic predispositions, and baseline metabolic profiles may alter susceptibility to NAFLD. Fourth, dietary assessment tools varied widely, from self-reported frequency to structured food frequency questionnaires, which may further introduce measurement error and misclassification bias. These inter-study variations highlight the need for more stratified analyses and standardized methodologies in future investigations to better account for potential effect modifiers and improve comparability across studies. (2) Observational design: the reliance on observational studies precludes causal inference. Reverse causation (e.g., individuals with NAFLD altering their diet) cannot be ruled out, though sensitivity analyses and the use of prospective cohort studies partially mitigate this concern. (3) Dietary assessment: most studies used self-reported questionnaires, which are prone to recall bias and misclassification of fast food intake. Future studies should incorporate objective measures (e.g., biomarkers or food frequency records). (4) Generalizability: the included studies were conducted in diverse regions (e.g., Lebanon, Spain, USA), but the majority focused on adults. The extrapolation of findings to children or specific ethnic groups (e.g., East Asians, who may have higher NAFLD susceptibility) remains uncertain. (5) Publication bias: while funnel plots showed no significant asymmetry, the possibility of unpublished negative studies cannot be entirely dismissed.

Furthermore, recent high-quality evidence supports a broader view on the relationship between dietary patterns and metabolic health outcomes, including NAFLD. For instance, an umbrella review by Lane et al. (23) synthesized meta-analyses and found that greater exposure to ultra-processed foods is consistently associated with increased risks of metabolic disorders, including obesity and type 2 diabetes, both of which are major risk factors for NAFLD. Additionally, data from a UK population-based study by Madruga et al. (24) highlighted that over 56% of dietary energy intake derives from ultra-processed foods, underscoring their dominant role in contemporary eating patterns. However, not all studies concur. Some reports have found no significant association between fast food or ultra-processed food intake and NAFLD, likely due to variation in diagnostic tools, sample demographics, and cultural dietary differences (8, 13, 16). Moreover, the predominance of data from Western or high-income countries in our analysis (e.g., USA, UK, Spain) limits generalizability to underrepresented regions such as Africa and South America. To provide a more balanced and comprehensive perspective, future studies should include diverse geographical settings and socio-economic backgrounds. It is also essential to integrate findings from recent narrative and systematic reviews on broader dietary patterns and liver health. Examples include recent reviews examining the associations between NAFLD and various dietary patterns, such as the Mediterranean diet, plant-based dietary interventions, and dietary classifications based on the NOVA system (25–30). Additionally, as most included studies relied on self-reported dietary intake, recall bias and misclassification of fast food exposure cannot be ruled out, which may have attenuated or inflated the observed associations.

In conclusion, this meta-analysis provides robust evidence that fast food consumption is associated with increased NAFLD and obesity risk. The findings highlight the need for public health interventions targeting dietary patterns to reduce NAFLD burden. However, heterogeneity in study design and methodology underscores the importance of standardized approaches in future research to validate these associations and inform precision prevention strategies. To enhance real-world applicability, these findings warrant the implementation of specific public health strategies. For example, regulatory policies such as mandatory front-of-package labeling, taxation on ultra-processed foods, and restrictions on fast food advertising to children may help reduce consumption. Additionally, national dietary guidelines should emphasize limiting fast food intake and promote the adoption of whole-food, plant-based dietary patterns. Public health campaigns and educational programs targeting high-risk populations can further support behavior change and reduce NAFLD prevalence.

## Data Availability

The original contributions presented in the study are included in the article/Supplementary material, further inquiries can be directed to the corresponding author.
