# Supplementary material for: Fast food consumption and risk of non-alcoholic fatty liver disease: a systematic review and meta-analysis
Source: Front Public Health. 2025 Jul 30;13:1600826. doi: 10.3389/fpubh.2025.1600826 (PMC12343633; doi:10.3389/fpubh.2025.1600826)
Supplement: Supplementary file 1 [file Table_1.docx]

# **Supplementary File 1:** Detailed NOS quality assessment for included studies

| Study | Case selection (/4) | Comparability (/2) | Outcome indicators (/3) | Total score (/9) |
| --- | --- | --- | --- | --- |
| Fakhoury-Sayegh et al., 2017 | 4 | 2 | 2 | 8 |
| García et al., 2025 | 4 | 2 | 3 | 9 |
| Kalafati et al., 2019 | 4 | 1 | 2 | 7 |
| Kardashian et al., 2023 | 4 | 2 | 2 | 8 |
| Liu et al., 2023 | 4 | 1 | 2 | 7 |
| Saha et al., 2022 | 4 | 2 | 2 | 8 |
| Talenezhad et al., 2022 | 4 | 1 | 2 | 7 |
| Zhang et al., 2024 | 4 | 2 | 2 | 8 |
| Zhao et al., 2024 | 4 | 1 | 2 | 7 |
